# Limited Metabolic Effect of the CREBRF^R457Q^ Obesity Variant in Mice

**DOI:** 10.3390/cells11030497

**Published:** 2022-01-31

**Authors:** Louise K. Metcalfe, Peter R. Shepherd, Greg C. Smith, Nigel Turner

**Affiliations:** 1 Department of Pharmacology, School of Medical Sciences, UNSW Sydney, Sydney, NSW 2052, Australia; l.metcalfe@unsw.edu.au (L.K.M.); g.smith@unsw.edu.au (G.C.S.); 2 Maurice Wilkins Centre for Molecular Biodiscovery, Auckland 1010, New Zealand; peter.shepherd@auckland.ac.nz; 3 Department of Molecular Medicine & Pathology, University of Auckland, Auckland 1023, New Zealand

**Keywords:** *CREBRF*, gene variant, obesity, glucose homeostasis, energy metabolism

## Abstract

The Arg457Gln missense variant in the *CREBRF* gene has previously been identified as driving excess body weight in Pacific/Oceanic populations. Intriguingly, Arg457Gln variant carriers also demonstrate paradoxical reductions in diabetes risk, indicating that the gene has a critical role in whole-body metabolism. To study the function of this variant in more detail, we generated mice on an FVB/N background with the *Crebrf* Arg458Gln variant knocked in to replace the endogenous *Crebrf*. The whole-body metabolic phenotype was characterized for male and female mice on a regular chow diet or an 8-week high-fat challenge. Regular assessment of body composition found that the *Crebrf* variant had no influence on total body weight or fat mass at any time point. Glucose tolerance tests demonstrated no obvious genotype effect on glucose homeostasis, with indirect calorimetry measures of whole-body energy expenditure likewise unaffected. Male chow-fed variant carriers displayed a trend towards increased lean mass and significantly reduced sensitivity to insulin administration. Overall, this novel mouse model showed only limited phenotypic effects associated with the *Crebrf* missense variant. The inability to recapitulate results of human association studies may invite reconsideration of the precise mechanistic link between CREBRF function and the risks of obesity and diabetes in variant allele carriers.

## 1. Introduction

Obesity is one of the most significant contemporary health issues, due both to its sharply rising global prevalence and the substantial number of adverse health problems that are associated with the obese state. While reduced physical activity and overconsumption of calorie-dense foods are no doubt major precipitates for the increased obesity rates worldwide, there is also intense interest in discovering genetic variants that may predispose individuals to weight gain and the associated co-morbidities of obesity. A recent genome-wide association study on a Samoan population identified a novel single nucleotide polymorphism (SNP) in the *CREBRF* gene (rs373863828, Arg457Gln), which was strongly linked with body mass index (BMI) [1]. The missense variant, which is located at a highly conserved position within the *CREBRF* gene, produced significant dose-dependent increases in obesity risk [1]. Strikingly, occurrence of this Arg457Gln missense variant is tightly restricted by descent and geography. It is present in all surveyed Polynesian populations, with reported minor allele frequencies as high as 0.259 in the Samoan cohort, but comparatively scarce even among the geographically close Melanesian and Micronesian populations and virtually non-existent in individuals outside the Pacific Islands [1,2,3,4,5,6].

While the heightened obesity risk is primarily underlain by measured increases in BMI, the variant has additionally been positively associated with increased body fat percentage, as well as waist and hip circumference [1,2,3]. More recently, associations with increased height and fat-free mass have also been described [6,7,8,9,10]. Intriguingly, carriers of the missense variant have a reported reduction in the risk of type 2 and gestational diabetes [1,5,11]. This phenotype is quite paradoxical, given the well-documented correlation between obesity and diabetes. The reduction in diabetes risk, however, is retained even following adjustment for BMI and has been attributed to (undefined) effects of the variant on glucose homeostasis, which cause significantly lower fasting blood glucose levels operating independently of insulin sensitivity or secretion [1,3,5]. Of note, carriers of the missense variant seem to develop their phenotypic differentiation early in life, with effects on weight, height, and waist circumference seen also in childhood [1,6,8,9,10].

The extant literature cannot yet provide definitive answers regarding either the strong predisposition towards obesity or the protection against type 2 diabetes (T2D) which are associated with the missense variant. Nor is it clear whether these two facets interact or operate independently. The CREBRF protein is primarily recognized to negatively regulate activity of the stress-responsive transcription factor CREB3, which along with CREBRF is involved in the maintenance of ER–Golgi homeostasis and in glucocorticoid and hypothalamic-pituitary-adrenal axis stress signalling [12,13,14,15,16]. CREBRF has further been implicated in cellular and organismal bioenergetics as a starvation response factor, conferring protection against nutrient deprivation [1,16,17]. This seems relevant, but mechanistic links to the variant phenotype at a systemic and tissue level remain uncertain. To address this in more detail, the present study sought to examine the metabolic phenotype of a novel knock-in (KI) mouse model for the *Crebrf* missense variant, with the underlying hypothesis that these animals would present a phenotype similar to human carriers of the *CREBRF* missense variant. Heterozygous (HET) and homozygous (HOM) carriers of both sexes underwent comprehensive metabolic characterization on a standard laboratory chow diet and in response to a high-fat, high-sugar diet. Mice carrying the *Crebrf* missense variant displayed limited phenotypic differences to their wild-type (WT) counterparts, indicating that the reported effects of the *CREBRF* Arg457Gln variant in humans involve more complex metabolic interactions than can be simply explained by the presence of the variant alone.

## 2. Materials and Methods

### 2.1. Animals

*Crebrf* Arg458Gln gene variant mice were generated on an FVB/N background by the MEGA Gene Engineering Facility (Moss Vale, NSW, Australia) via a CRISPR/Cas9 approach, with genotyping details provided in [7]. Age-matched male and female WT, HET, and HOM mice were housed from eight weeks of age at 22 ± 1 °C with a controlled 12:12 h light-dark cycle and had ad libitum access to water and diets. After four weeks (i.e., aged 12 weeks) on a standard control “chow” diet (71% of calories from carbohydrate, 8% calories from fat, 21% calories from protein, ~3 kcal/g; Gordon’s Specialty Stock Feeds, Yanderra, NSW, Australia), mice were randomly allocated to remain on the chow diet or to receive an in-house-made high fat diet (HFD) (45% calories from fat; 4.7 kcal/g; [18]) ad libitum for eight weeks (i.e., until 20 weeks post-birth). Plasma and tissue samples were collected from mice either in a fed state or following a 15–16 h overnight fasting period.

All animal experiments were approved by the UNSW Animal Care and Ethics Committee (ACEC 15/48B, 18/78A) and followed guidelines issued by the National Health and Medical Research Council of Australia.

### 2.2. Body Composition and Energy Expenditure

Body composition (lean mass and fat mass) was measured using the EchoMRI-900 Body Composition Analyzer (EchoMRI Corporation Pty Ltd., Singapore) in accordance with the manufacturer’s instructions.

The heat production and respiratory exchange ratio (RER) of individual mice were measured using an Oxymax indirect calorimeter (Columbus Instruments, Columbus, OH, USA) as previously described [18]. Data were collected over a 24 h period (12 h light/dark cycle, 0700–1900 h), following a 16 h acclimatization period.

### 2.3. Glucose and Insulin Tolerance

Mice were fasted for 5 h and then given either glucose via oral gavage (3 g/kg lean mass) or insulin via intraperitoneal injection (1.0 U/kg lean mass). Blood glucose levels were monitored using an Accu-Chek glucometer (Roche Diagnostics, Castle Hill, NSW, Australia). For the glucose tolerance test (GTT), performed in mice aged 16 weeks, glucose was analyzed at baseline and at 15, 30, 60, and 90 min post gavage. For the insulin tolerance test (ITT), performed in mice aged 17 weeks, glucose levels were determined at baseline and at 10, 20, 30, 45, and 60 min post injection. During the GTT, blood was collected from the tail tip prior to and at 15, 30, and 60 min following glucose gavage to measure plasma insulin using an Ultrasensitive Mouse Insulin ELISA Kit (Crystal Chem, Elk Grove Village, IL, USA) in accordance with manufacturer’s instructions.

### 2.4. Determination of Glycogen Content

Frozen tissue (15–30 mg) was digested in 200 μL of 1 M potassium hydroxide at 70 °C for 1 h with regular mixing. Precipitation of glycogen was facilitated by the addition of 1.75 mL 95% ethanol and 75 μL saturated sodium sulphate (Na_2_SO_4_) followed by incubation at −80 °C for 1 h and centrifugation at 13,000× *g* for 10 min at 4 °C. The glycogen pellet was dissolved in 200 μL water and an additional ethanol precipitation was performed. The final pellet was air-dried at RT and dissolved in 0.3 mg/mL amyloglucosidase (Sigma) solution prepared in sodium acetate buffer (0.25 M, pH 4.75) and incubated at 37 °C overnight. Glucose concentration was measured using a colorimetric assay (Infinity Glucose (Ox), Thermo).

### 2.5. Tissue and Plasma Lipids Analyses

Lipids were extracted from liver and quadriceps tissue using standard methods [19]. Tissue and plasma triglyceride contents were determined using a colorimetric assay kit (Triglycerides GPO-PAP, Roche Diagnostics, Castle Hill, NSW, Australia). Similarly, plasma non-esterified fatty acids (NEFAs) were measured using a colorimetric kit (NEFA C kit, Wako Chemicals, Osaka, Japan).

### 2.6. Statistical Analysis

Data were analyzed by a standard or repeated measures 2-way ANOVA or 3-way ANOVA, with genotype, diet, and feeding state as the relevant factors where appropriate. Where a significant main effect was identified for any of the factors by the ANOVA, a Dunnett’s or Sidak’s post hoc test was conducted to determine differences between groups. Male and female mice were analyzed separately due to known differences in body composition and susceptibility to diet-induced metabolic dysfunction [20,21]. Values are presented as mean ± SEM, with statistical significance accepted at *p* < 0.05. Statistical analysis was performed in GraphPad Prism software (Prism 8, Version 8.0.2, GraphPad Software, San Diego, CA, USA).

## 3. Results

### 3.1. Body Composition

**Figure 1:**
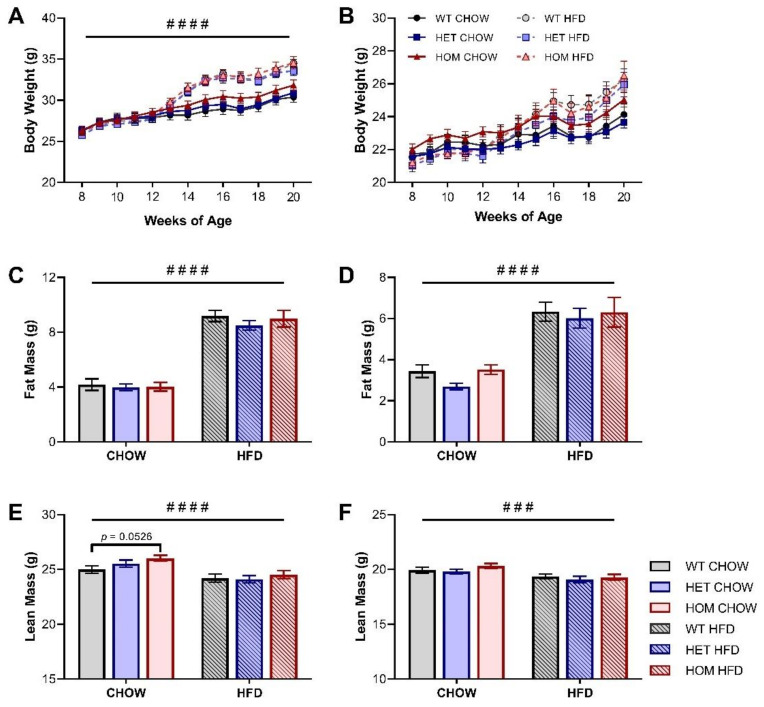
Body composition of *Crebrf* R458Q variant mice. (**A**,**B**) Body weight of (**A**) male and (**B**) female mice throughout the study (HFD began at 12 weeks of age). (**C**,**D**) Total fat mass at 20 weeks of (**C**) male and (**D**) female mice. (**E**,**F**) Total lean mass at 20 weeks of (**E**) male and (**F**) female mice. Data are presented as mean ± SEM, *n* = 17–24. ### *p* < 0.001, #### *p* < 0.0001 main effect of diet by RM (**A**,**B**) or ordinary (**C**–**F**) two-way ANOVA; *p* = 0.0526 WT chow vs HOM chow by Dunnett’s multiple comparisons.

The human variant allele has been repeatedly associated with increased BMI and/or adiposity measures [1,2,3,4], however the body weight of the HOM and HET KI mice on the chow diet was not significantly different from WT mice across the course of the study for either sex (Figure 1A,B, Table 1). Total body fat mass and the weight of individual adipose depots, measured at the conclusion of the feeding regime, were similarly unaffected by genotype for both male and female mice (Figure 1C,D, Table 1). There was no genotype difference in the weight of several key organs (Table 1), however, EchoMRI lean mass measurements showed that male HOM chow-fed mice trend towards greater lean mass (*p* = 0.0526) than their WT counterparts at 20 weeks of age (Figure 1E). The chow-fed HET animals demonstrated an intermediate increase in lean mass suggestive of a linear or dose-dependent trend between these three chow-fed genotype groups (Figure 1E). Female mice did not display any genotype-dependent differences in lean mass or the weight of individual organs (Figure 1F, Table 1).

To examine the effects of diet on body composition, a HFD was introduced to half the cohort at 12 weeks of age and maintained for 8 weeks. Both male and female sexes experienced the expected significant increases in body weight, total fat content, and adipose depot size following HFD feeding, but there was no diet-induced development of a genotype effect (Figure 1A–D, Table 1). Nor was any genotype effect on lean mass identified in HFD-fed mice of either sex (Figure 1E,F).

### 3.2. Glucose and Insulin Tolerance

**Figure 2:**
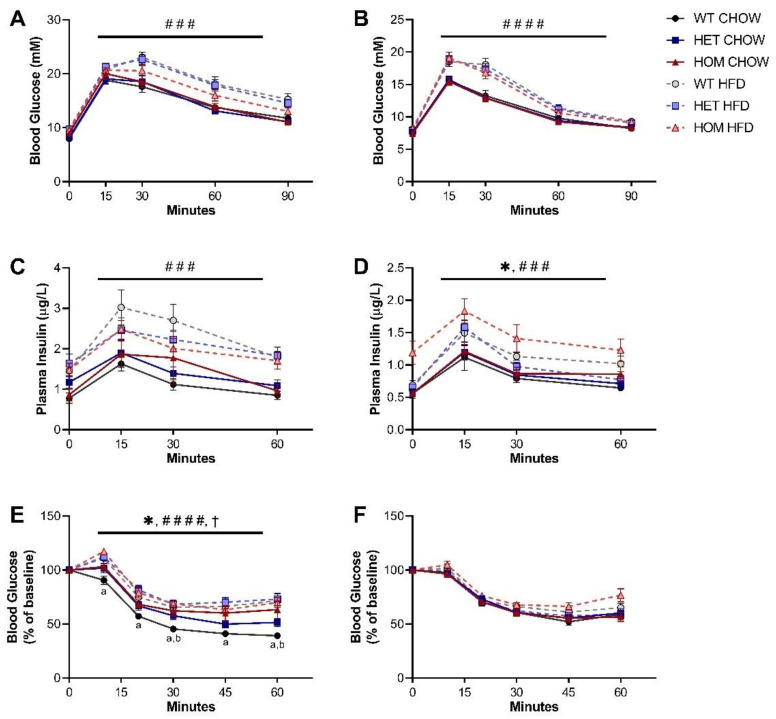
Glucose and insulin tolerance in *Crebrf* R458Q variant mice. (**A**,**B**) Blood glucose response to glucose administration for 16-week (**A**) male and (**B**) female animals (*n* = 13–19). (**C**,**D**) Plasma insulin levels during oGTT for (**C**) males and (**D**) females (*n* = 8–16). (**E**,**F**) Blood glucose response to insulin administration, presented as percentage of baseline fasting levels at the time of insulin administration, in 17-week (**E**) males and (**F**) females (*n* = 10–18). Data are presented as mean ± SEM. ### *p* < 0.001, #### *p* < 0.0001 main effect of diet, * *p* < 0.05 main effect of genotype, † *p* < 0.05 main effect of interaction between diet and genotype by RM three-way ANOVA. a *p* < 0.05 WT chow vs. HOM chow, b *p* < 0.05 WT chow vs. HET chow by Dunnett’s multiple comparisons.

To determine if the R458Q variant impacted glycaemic control in the KI mice, an oral GTT was performed at the 16-week timepoint (4 weeks into the HFD feeding regime). Glucose clearance of both sexes was significantly impaired by HFD feeding but was unaffected by genotype (Figure 2A,B). Plasma insulin levels during the GTT were likewise unchanged by genotype in male mice, but in females there was a significant main effect of genotype to cause mildly increased insulin levels compared to WT animals (Figure 2C,D). HFD feeding significantly heightened plasma insulin levels for both sexes, while there was no significant interaction between genotype and diet observed for either sex.

We further assessed whole-body insulin sensitivity via intraperitoneal ITT. On a chow diet, male HOM mice exhibited a significantly less pronounced fall in glucose levels post-injection than was seen in WT counterparts (Figure 2E). This apparent comparative reduction in sensitivity to insulin administration was not overtly dose-dependent, but chow-fed HET males did display an intermediate response, positioned between WT and HOM groups. The genotype effect was absent in HFD-fed male mice, although analysis of both diet groups showed significant main effects of genotype as well as of interaction between genotype and diet. Unexpectedly, the insulin sensitivity of male HOM mice also appeared unaffected by HFD feeding, with the glucose response of mice carrying two copies of the variant not different between diet groups. In contrast, insulin tolerance of female mice was unchanged by either genotype or diet (Figure 2F).

### 3.3. Indirect Calorimetry

**Figure 3:**
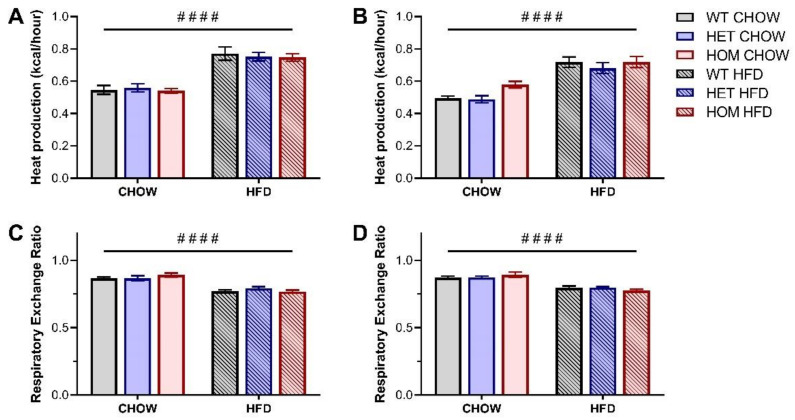
Whole-body energy expenditure in *Crebrf* R458Q variant mice at 18 weeks. (**A**,**B**) Heat production, normalized to lean mass, in (**A**) male and (**B**) female animals. (**C**,**D**) Respiratory exchange ratio (RER) in (**C**) male and (**D**) female animals. Values represent an average over a 24-h data collection period (12 h light and 12 h dark phase) presented as mean ± SEM, *n* = 6–8. #### *p* < 0.0001 main effect of diet by ordinary two-way ANOVA.

Given that CREBRF has been previously associated with altered cellular bioenergetics [1], we additionally performed indirect calorimetry to determine whether the R458Q variant impacted measures of whole-body energy metabolism and expenditure in our knock-in mouse model. Whole-body energy expenditure (as measured by heat production) was significantly increased for HFD-fed male and female mice compared to their chow-fed counterparts (Figure 3A,B), while as expected HFD-fed mice exhibited a significantly lower respiratory exchange ratio (RER) than their chow-fed counterparts (Figure 3C,D). No differences were observed between genotypes for either parameter.

### 3.4. Biochemical Analyses

Plasma and tissue biochemical analyses (Table 2) were performed on samples taken from WT and HOM mice in either the fed state or following overnight fasting, which aimed to induce conditions more likely to stimulate activity of the starvation factor CREBRF [1,22]. Blood glucose levels were significantly depressed in response to fasting in both sexes under both dietary conditions, but there was no significant genotype effect on glucose levels in either the fed or fasted state. The presence of the R458Q variant likewise had no effect on plasma insulin levels in both male and female mice, but the expected main effects for a decrease in insulin due to fasting and increase in response to high fat feeding were apparent. Plasma triglyceride was unaltered by genotype in male mice. In females, however, the R458Q variant was associated with a decrease in plasma triglyceride levels (Table 2). In contrast, circulating NEFAs in male mice were increased in HOM animals, with this change largely driven by a more pronounced increase in fasting NEFA levels in mice carrying the R458Q variant. Female mice did not present the same pattern, with a clear fasting effect, but the absence of any difference as a result of genotype.

**Figure 4:**
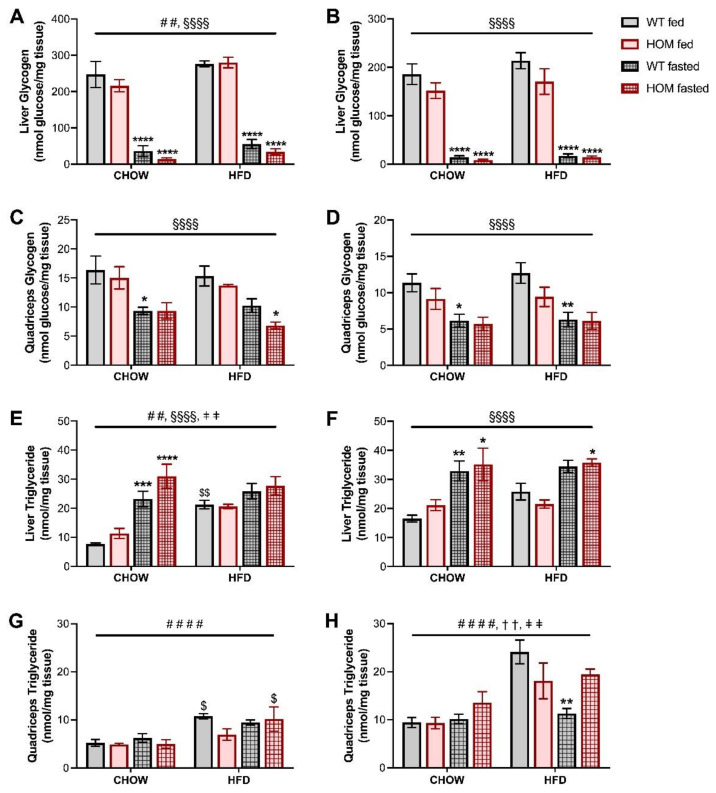
Tissue nutrient homeostasis in fed or overnight-fasted *Crebrf* R458Q variant mice at 20 weeks. (**A**–**D**) Glycogen content in liver tissue of (**A**) male and (**B**) female animals, and in quadriceps tissue of (**C**) males and (**D**) females. (**E**–**H**) Triglyceride content in liver tissue of (**E**) male and (**F**) female animals, and in quadriceps tissue of (**G**) males and (**H**) females. Data are presented as mean ± SEM, *n* = 5–6. ## *p* < 0.01, #### *p* < 0.0001 main effect of diet; §§§§ *p* < 0.0001 main effect of fasting; †† *p* < 0.01 main effect of interaction between genotype and fed state; ǂǂ *p* < 0.01 main effect of interaction between fed state and diet by three-way ANOVA. $ *p* < 0.05, $$ *p* < 0.01 effect of high fat diet vs. corresponding chow-fed group; * *p* < 0.05, ** *p* < 0.01, *** *p* < 0.001, **** *p* < 0.0001 effect of fasting vs. corresponding fed group within the same diet by Sidak’s post hoc test.

As a broad measure of tissue nutrient homeostasis, glycogen and triglyceride contents were measured in both liver and muscle. Glycogen stores in both tissues were as expected significantly depleted by the overnight fasting for all mice, with a greater decline observed in the liver (Figure 4A–D). The presence of the *Crebrf* Arg457Gln variant in male mice did not significantly impact glycogen content in either tissue, but in the fasted state there was a trend for variant-induced reductions. There was an overall trend (*p* = 0.0565) for female liver glycogen to be reduced in HOM mice, and unlike the males, this was more prominent in animals in the fed state (Figure 4B); the pattern was reflected also in quadriceps glycogen content (Figure 4D). Hepatic triglyceride accumulation in chow-fed male mice was significantly increased by fasting, with a trend (*p* = 0.07) for a main effect of the variant on this parameter (Figure 4E). HFD blunted the fasting and genotype-induced triglyceride changes (Figure 4E). Female mice exhibited a similar overall pattern in hepatic triglyceride levels among both chow- and HFD-fed animals, but without significant genotype effects (Figure 4F). Muscle triglycerides were predominantly influenced by the HFD in both male and female mice (Figure 4G,H). Female muscle displayed a significant interaction between genotype and fed state, and between fed state and diet, with the overnight fast associated with a relative decrease in triglyceride levels in HFD-fed WT animals but not their HOM counterparts.

## 4. Discussion

While there is an obvious link between the growing obesity epidemic and environmental factors, there has also been great interest in searching for genetic factors that may contribute to this condition. To date the most prominent genetic variant linked with excess body weight is the recently identified R457Q variant in the *CREBRF* gene, a gene variant with a presence restricted to the Pacific Islands [1,2,3]. Despite these reports, in the current study, mice with this variant knocked in did not display a metabolic phenotype consistent with human carriers of this variant, but rather exhibited differences in only a limited number of metabolic traits compared to their wildtype counterparts.

Over the course of the study, HOM mice were indistinguishable from WT animals when assessed for changes in body weight and fat mass, despite excess body weight being the most overt and well-reported effect of the variant in human studies. This lack of difference between the genotypes persisted when mice were placed on a HFD to provide a more obesogenic environment. Precise measures of body fat content, such as those which we have been able to determine for our KI mouse model, are rarely reported in human studies of the R457Q variant. Human studies most commonly utilize BMI as the chosen measure of obesity, reporting (with few exceptions) significant increases due to the minor allele. The use of BMI is not without its detractors. Most relevant here is the dubious nature of its ability to consistently represent comparative adiposity between ethnicities via uniform cut-off values developed for a specific population [23]. At a given BMI, Pacific Islanders have been reported to possess a higher ratio of lean mass to fat mass (i.e., less body fat mass) than, for example, Asian Indians or Europeans [24,25]. It has also been suggested (again with reference to Pacific Island populations) that BMI cut-offs should be based on associated comorbidities, not body composition alone [26]. Given the discrepancies between chosen methods of assessment, an exact comparison between mouse and human phenotypes are perhaps not wholly straightforward**.** For adiposity measures beyond BMI, Minster et al. [1] attributed increased body fat percentage to the missense variant, and along with some subsequent studies also reported increased waist and hip circumference in carriers of the variant [1,2,3]. A more recent clinical study, however, indicated that a cohort of Maori and Pacific pregnant women with obesity did not differ in BMI, waist circumference, or gestational weight gain between variant carriers and non-carriers [11]. These disparate findings imply that there is likely a more complex phenotypic effect of the missense variant than is otherwise yet apparent from published literature, or indeed within our mouse model.

We have identified that the missense variant resulted in a trend for increased lean mass in male but not female KI mice. Although this effect cannot be directly linked to any specific organ at present, no similar such effect has been reported in adult human studies to date. Arslanian et al. [10] did however report that Samoan infants carrying the variant exhibit increased lean mass and bone mass accretion, without any effects on BMI. These findings are consistent with trending increase in lean mass in our KI male mice and may indicate that some underlying correlations in mechanism could exist. Fat-free mass in human variant carriers outside infancy has not been investigated but, if increased, could potentially distort simple BMI measurements. Likewise, association of the missense variant with body composition or obesity measures may also be influenced by the more recently observed effects to increase human height as well as length in our KI male mice, as previously published [4,5,7,8,9]. Dual effects on fat-free mass and height imply that the missense variant could produce a growth phenotype, resulting in greater overall body size, which is present in human carriers, albeit partially masked by Pacific Island-typical obesity. In keeping with these observations, animal models studying the WT CREBRF are also shown to experience growth-related effects, with mice and flies lacking the protein being lighter and smaller [26,27].

The second major facet of the known *CREBRF* missense variant phenotype, namely a decreased diabetes risk [1], similarly seems to be poorly recapitulated in the murine model. The missense variant in our KI mice had limited overall effect on glucose clearance during a glucose tolerance test in both female and male mice. Insulin tolerance testing in the female and male KI mice revealed that the blood glucose response to insulin administration is largely unaffected by the missense variant, and if anything, hints at an induction of insulin resistance by the variant in chow-fed male animals, where there was an attenuation in insulin-induced effects. Taken together, the combination of minimally altered glucose homeostasis, unchanged basal levels of glucose or insulin, and the blunted response to insulin which is seen in our male KI mice entirely fails to provide any overt reproduction of the human phenotype.

Comparatively few studies report on specific metabolic factors which may contribute to the human R457Q missense variant effects on diabetes. Minster et al. [1] reported significantly lower fasting glucose levels (0.09 mM per variant allele) even in non-diabetic subjects, with the effect being more pronounced after adjustment for BMI. No consistent correlations with either fasting insulin levels or HOMA-IR have been reported. Although Hanson et al. [5] found an association with higher HOMA-B, this implied enhancement of β-cell capacity for compensatory insulin secretion was attenuated when adjusted for BMI. The association with increased glucose-stimulated insulin release more recently identified in normoglycemic overweight/obese men of Maori and Pacific ancestry was however significant for early phase insulin secretion consistent with T2D prevention [28]. Others have recently speculated that, in humans, the protective effects against type 2 and gestational diabetes may derive from the greater (height-related) muscle and bone mass that has been reported in variant carriers and predicted to enhance glucose disposal [10,11]. The similarly increased lean mass and length of our male KI mice without positive effects on glucose homeostasis does not align with this tenet, and deeper study would be needed to untangle the potential origins of the contrast between phenotypes displayed by mouse and human missense variant carriers. Indeed, it is possible that a more challenging or different diet regimen, or an experimental model of induced T2D, might be required to reveal an underlying genotype effect in the KI mice (if one exists).

The initial theory proposed by Minster et al. [1] to explain the phenotype seen in human variant carriers would expect the *CREBRF* variant to produce a “thriftier” balance between energy storage and expenditure. Our KI mice, however, did not present any whole-body impact of genotype on either heat production or respiratory exchange ratio measures when examined via indirect calorimetry. The concept of the thrifty genotype is integrated with the fasting state, as a theorised, evolutionarily adapted mechanism to survive prolonged nutrient deprivation [29]. Both wildtype and variant CREBRF have been shown in vitro to promote cell survival during nutritional stress [1]. We therefore examined our KI mice following overnight fasting using biochemical measures as proxies for energy storage. Taken together, the combined fasting-induced trends in tissue glycogen and lipid contents, as well as plasma NEFA levels, imply that the missense variant may indeed have some role in regulating energy homeostasis during periods of nutrient deprivation. The hint towards an exaggerated fasting response, observed in male mice carrying two copies of the variant, is perhaps not sufficiently definitive to draw firm conclusions based on the current sample alone. Given that both the wild-type and variant *CREBRF* are induced by starvation, it does however seem logical that these conditions would promote greater activity and thus more overt genotype differentiation [1,27]. The WT CREBRF response to nutrient deprivation follows mammalian target of rapamycin complex 1 (mTORC1)-independent cell stress signalling, to which it is particularly sensitive, in addition to signals mediated by mTORC1 inhibition [1,22]. Indeed, the *Drosophila* ortholog mediates the downstream transcriptional response triggered by TORC1 inhibition, and its absence renders flies more sensitive to starvation [27]. A missense variant-induced differentiation in the fasting response, on tissue or organismal scales, may also contribute to effects on glucose disposal or insulin response, if not potentially longer-term effects on bioenergetics. Given the relatively minor effect size in our KI mice, however, speculation may be strengthened by a more severe deprivation protocol than the overnight fast used in the current study.

Kanshana et al. [30] have recently reported extensive phenotypic characterization of their knock-in *Crebrf* R458Q variant mouse model, generated on a C57BL/6J background. Grounded in the known literature associations with starvation and mTORC1 inhibition, as described above, these animals were assessed across multiple nutritional conditions including low- or high-fat diets, fasting and refeeding, prolonged nutritional stress, or pharmacological mTORC1 inhibition. Contrary to the current study’s evidence of a potential murine missense variant effect during fasting, however, Kanshana et al. [30] concluded that the R458Q variant did not influence energy or glucose homeostasis in their mouse model even when exposed to more extreme nutrient deprivation, with no effect on length or lean mass. The disparity between these KI mouse models, and between sexes in the current study, demonstrates that manifestation of the variant phenotype can be disrupted not only by species translation but also by mouse strain and sex, or potentially by a less tangible factor of handling, housing, study design, or location. Housing temperature, for example, can distinctly influence energy homeostasis [31], and housing KI mice closer to their thermoneutral temperature, perhaps more akin to the tropical Pacific environment where the human phenotype primarily occurs, could potentially impact the phenotype of these animals. Overall, the factors underlying any robustness (or, perhaps, fragility) of CREBRF genotype effects across differing models and conditions, might aid the development of suitable preclinical models to investigate the importance of this variant.

In conclusion, comprehensive metabolic phenotyping of mice harboring the *Crebrf* R458Q variant revealed only limited effects of the mutation, which did not reproduce either the increased obesity or decreased diabetes risk reported in human variant carriers. This may potentially be influenced by the background of the mouse strain used in the current study, given the importance of mouse strain in the differentiation of metabolic responses [32,33]. Translational discrepancies may also, however, reflect the more complex and multifactorial nature of the metabolic interactions which are present in humans. The influence of these interactions on the CREBRF missense variant likely necessitates a more challenging protocol to reproduce or understand the phenotypic consequences in this mouse model.

## Figures and Tables

**Table 1:** Tissue weights in CHOW- and HFD-fed *Crebrf* R458Q variant mice at 20 weeks.

Sex	Tissue	CHOW			HFD			*p*-Values		
		WT	HET	HOM	WT	HET	HOM	G	D	G × D
**Male**	BW (g)	30.1 ± 0.83	31.0 ± 0.70	31.3 ± 1.04	34.3 ± 0.91	33.8 ± 0.60	34.7 ± 1.13	ns	<0.0001	ns
	eWAT (% BW)	1.64 ± 0.23	1.66 ± 0.13	1.86 ± 0.26	3.78 ± 0.11	3.67 ± 0.14	3.54 ± 0.17	ns	<0.0001	ns
	iWAT (% BW)	0.61 ± 0.06	0.66 ± 0.04	0.77 ± 0.08	1.33 ± 0.08	1.11 ± 0.05	1.39 ± 0.16	ns	<0.0001	ns
	Liver (% BW)	4.65 ± 0.13	4.75 ± 0.14	4.70 ± 0.15	4.21 ± 0.09	4.16 ± 0.16	4.08 ± 0.11	ns	<0.0001	ns
	Heart (% BW)	0.46 ± 0.01	0.46 ± 0.01	0.45 ± 0.02	0.41 ± 0.02	0.41 ± 0.01	0.41 ± 0.02	ns	<0.001	ns
	Kidneys (% BW)	1.54 ± 0.05	1.53 ± 0.02	1.45 ± 0.03	1.25 ± 0.04	1.28 ± 0.03	1.26 ± 0.04	ns	<0.0001	ns
**Female**	BW (g)	24.6 ± 0.94	23.8 ± 0.60	24.8 ± 0.83	27.3 ± 0.88	26.3 ± 1.10	25.9 ± 0.83	ns	<0.01	ns
	pWAT (% BW)	1.27 ± 0.25	0.98 ± 0.18	1.57 ± 0.21	3.31 ± 0.30	3.02 ± 0.36	2.71 ± 0.48	ns	<0.0001	ns
	iWAT (% BW)	0.83 ± 0.08	0.67 ± 0.06	0.78 ± 0.06	1.31 ± 0.05	1.22 ± 0.07	1.25 ± 0.09	ns	<0.0001	ns
	Liver (% BW)	4.99 ± 0.11	5.03 ± 0.11	4.95 ± 0.13	4.33 ± 0.04	3.99 ± 0.08	4.13 ± 0.17	ns	<0.0001	ns
	Heart (% BW)	0.50 ± 0.02	0.48 ± 0.01	0.46 ± 0.01	0.41 ± 0.01	0.47 ± 0.03	0.44 ± 0.03	ns	<0.01	ns
	Kidneys (% BW)	1.25 ± 0.03	1.25 ± 0.04	1.23 ± 0.02	1.05 ± 0.02	1.06 ± 0.01	1.09 ± 0.05	ns	<0.0001	ns

Data are presented as mean ± SEM, *n* = 8–11. Statistics by ordinary two-way ANOVA. BW = body weight; eWAT = epididymal white adipose tissue; HFD = high fat diet; iWAT = inguinal white adipose tissue; pWAT = periovarian white adipose tissue; G = main effect of genotype; D = main effect of diet; G × D = main effect of interaction between genotype and diet; ns = not significant.

**Table 2:** Circulating factors in CHOW- and HFD-fed *Crebrf* R458Q variant mice at 20 weeks.

Sex	Circulating Factor	Chow		HFD		*p*-Values			
		WT	HOM	WT	HOM	G	F	D	G × F
**Male**	Fed BG (mM)	8.52 ± 0.22	8.63 ± 0.13	8.47 ± 0.23	8.70 ± 0.28	ns	<0.0001	ns	ns
	Fasted BG (mM)	5.53 ± 0.27 ^§§§§^	5.99 ± 0.63 ^§§§§^	6.11 ± 0.23 ^§§§§^	6.44 ± 0.31 ^§§§§^				
	Fed Insulin (μg/L)	1.40 ± 0.17	1.68 ± 0.41	2.67 ± 0.50	2.44 ± 0.55	ns	<0.0001	<0.05	ns
	Fasted Insulin (μg/L)	0.51 ± 0.06	0.46 ± 0.04	0.58 ± 0.07 ^§§§^	0.59 ± 0.05 ^§§^				
	Fed TAGs (mM)	2.03 ± 0.20	2.08 ± 0.19	1.39 ± 0.14	1.44 ± 0.15	ns	ns	<0.01	ns
	Fasted TAGs (mM)	2.21 ± 0.29	1.66 ± 0.13	1.83 ± 0.18	1.80 ± 0.12				
	Fed NEFAs (mM)	0.40 ± 0.07	0.40 ± 0.08	0.38 ± 0.07	0.49 ± 0.05	<0.05	<0.0001	ns	ns
	Fasted NEFAs (mM)	0.77 ± 0.07	1.19 ± 0.14 *^,§§§§^	0.80 ± 0.09 ^§^	0.92 ± 0.13 ^§^				
**Female**	Fed BG (mM)	7.95 ± 0.18	8.54 ± 0.18	7.88 ± 0.23	8.18 ± 0.14	ns	<0.0001	ns	ns
	Fasted BG (mM)	5.30 ± 0.20 ^§§§§^	5.61 ± 0.38 ^§§§§^	5.55 ± 0.34 ^§§§§^	5.16 ± 0.27 ^§§§§^				
	Fed Insulin (μg/L)	0.63 ± 0.11 ^##^	0.76 ± 0.10	2.07 ± 0.53	1.04 ± 0.24	ns	<0.001	<0.01	ns
	Fasted Insulin (μg/L)	0.48 ± 0.13	0.51 ± 0.05	0.53 ± 0.06 ^§§§^	0.48 ± 0.04				
	Fed TAGs (mM)	2.55 ± 0.13	2.89 ± 0.25 ^#^	2.44 ± 0.26	1.90 ± 0.11	<0.05	ns	<0.0001	ns
	Fasted TAGs (mM)	3.52 ± 0.36 ^##^	2.70 ± 0.23 ^#^	2.19 ± 0.16	1.73 ± 0.15				
	Fed NEFAs (mM)	0.45 ± 0.09	0.42 ± 0.04	0.32 ± 0.10	0.48 ± 0.03	ns	<0.0001	ns	<0.05
	Fasted NEFAs (mM)	1.21 ± 0.12 ^§§§§^	1.00 ± 0.08 ^§§§^	1.13 ± 0.06 ^§§§§^	0.99 ± 0.09 ^§§^				

Data are presented as mean ± SEM, *n* = 5–11. * *p* < 0.05 vs. WT counterpart; **^§^** *p* < 0.05, **^§§^** *p* < 0.01, **^§§§^** *p* < 0.001, **^§§§§^** *p* < 0.0001 vs. fed state counterpart; **^#^** *p* < 0.05, **^##^** *p* < 0.01 vs. HFD counterpart by three-way ANOVA, Sidak’s post-hoc test. BG = blood glucose; NEFA = non-esterified fatty acid; TAG = triglyceride. G = main effect of genotype; F = main effect of feeding state; D = main effect of diet; G × F = main effect of interaction between genotype and feeding state; ns = not significant.

## Data Availability

The data presented in this study are available on request from the corresponding author.

## References

[1] Minster R.L., Hawley N.L., Su C.T., Sun G., Kershaw E.E., Cheng H., Buhule O.D., Lin J., Reupena M.S., Viali S. (2016). A thrifty variant in CREBRF strongly influences body mass index in Samoans. Nat. Genet..

[2] Naka I., Furusawa T., Kimura R., Natsuhara K., Yamauchi T., Nakazawa M., Ataka Y., Ishida T., Inaoka T., Matsumura Y. (2017). A missense variant, rs373863828-A (p.Arg457Gln), of CREBRF and body mass index in Oceanic populations. J. Hum. Genet..

[3] Krishnan M., Major T.J., Topless R.K., Dewes O., Yu L., Thompson J.M., McCowan L., de Zoysa J., Stamp L.K., Dalbeth N. (2018). Discordant association of the CREBRF rs373863828 minor allele with increased BMI and protection from type 2 diabetes in Māori and Pacific (Polynesian) people living in Aotearoa/New Zealand. Diabetologia.

[4] Lin M., Caberto C., Wan P., Li Y., Lum-Jones A., Tiirikainen M., Pooler L., Nakamura B., Sheng X., Porcel J. (2020). Population-specific reference panels are crucial for genetic analyses: An example of the CREBRF locus in Native Hawaiians. Hum. Mol. Genet..

[5] Hanson R.L., Safabakhsh S., Curtis J.M., Hsueh W.C., Jones L.I., Aflague T.F., Duenas Sarmiento J., Kumar S., Blackburn N.B., Curran J.E. (2019). Association of CREBRF variants with obesity and diabetes in Pacific Islanders from Guam and Saipan. Diabetologia.

[6] Berry S.D., Walker C.G., Ly K., Snell R.G., Atatoa Carr P.E., Bandara D., Mohal J., Castro T.G., Marks E.J., Morton S.M. (2018). Widespread prevalence of a CREBRF variant amongst Māori and Pacific children is associated with weight and height in early childhood. Int. J. Obes..

[7] Metcalfe L.K., Krishnan M., Turner N., Yaghootkar H., Merry T.L., Dewes O., Hindmarsh J.H., de Zoysa J., Dalbeth N., Stamp L.K. (2020). The Māori and Pacific specific CREBRF variant and adult height. Int. J. Obes..

[8] Carlson J.C., Rosenthal S.L., Russell E.M., Hawley N.L., Sun G., Cheng H., Naseri T., Reupena M.S., Tuitele J., Deka R. (2020). A missense variant in CREBRF is associated with taller stature in Samoans. Am. J. Hum. Biol..

[9] Oyama S., Duckham R.L., Arslanian K.J., Kershaw E.E., Strayer J.A., Fidow U.T., Naseri T., Hawley N.L. (2021). Body size and composition of Samoan toddlers aged 18–25 months in 2019. Ann. Hum. Biol..

[10] Arslanian K.J., Fidow U.T., Atanoa T., Unasa-Apelu F., Naseri T., Wetzel A.I., Pomer A., Duckham R.L., McGarvey S.T., Strayer J.A. (2021). A missense variant in CREBRF, rs373863828, is associated with fat-free mass, not fat mass in Samoan infants. Int. J. Obes..

[11] Krishnan M., Murphy R., Okesene-Gafa K.A., Ji M., Thompson J.M., Taylor R.S., Merriman T.R., McCowan L.M., McKinlay C.J. (2020). The Pacific-specific CREBRF rs373863828 allele protects against gestational diabetes mellitus in Māori and Pacific women with obesity. Diabetologia.

[12] Penney J., Mendell A., Zeng M., Tran K., Lymer J., Turner P.V., Choleris E., MacLusky N., Lu R. (2017). LUMAN/CREB3 is a key regulator of glucocorticoid-mediated stress responses. Mol. Cell. Endocrinol..

[13] Penney J., Taylor T., MacLusky N., Lu R. (2018). LUMAN/CREB3 plays a dual role in stress responses as a cofactor of the glucocorticoid receptor and a regulator of secretion. Front. Mol. Neurosci..

[14] Martyn A.C., Choleris E., Gillis D.J., Armstrong J.N., Amor T.R., McCluggage A.R., Turner P.V., Liang G., Cai K., Lu R. (2012). Luman/CREB3 recruitment factor regulates glucocorticoid receptor activity and is essential for prolactin-mediated maternal instinct. Mol. Cell. Biol..

[15] Frahm K.A., Williams A.A., Wood A.N., Ewing M.C., Mattila P.E., Chuan B.W., Guo L., Shah F.A., O’Donnell C.P., Lu R. (2020). Loss of CREBRF reduces anxiety-like behaviours and circulating glucocorticoids in male and female mice. Endocrinology.

[16] Audas T.E., Li Y., Liang G., Lu R. (2008). A novel protein, Luman/CREB3 recruitment factor, inhibits luman activation of the unfolded protein response. Mol. Cell. Biol..

[17] Audas T.E., Hardy-Smith P.W., Penney J., Taylor T., Lu R. (2015). Characterisation of nuclear foci-targeting of Luman/CREB3 recruitment factor (LRF/CREBRF) and its potential role in inhibition of herpes simplex virus-1 replication. Eur. J. Cell Biol..

[18] Turner N., Bruce C.R., Beale S.M., Hoehn K.L., So T., Rolph M.S., Cooney G.J. (2007). Excess lipid availability increases mitochondrial fatty acid oxidative capacity in muscle: Evidence against a role for reduced fatty acid oxidation in lipid-induced insulin resistance in rodents. Diabetes.

[19] Folch J., Lees M., Stanley G.H. (1957). A simple method for the isolation and purification of total lipides from animal tissues. J. Biol. Chem..

[20] Reed D.R., Bachmanov A.A., Tordoff M.G. (2007). Forty mouse strain survey of body composition. Physiol. Behav..

[21] Rudnicki M., Abdifarkosh G., Rezvan O., Nwadozi E., Roudier E., Haas T.L. (2018). Female mice have higher angiogenesis in perigonadal adipose tissue than males in response to high-fat diet. Front. Physiol..

[22] Tiebe M., Lutz M., Tiebe D.S., Teleman A.A. (2019). Crebl2 regulates cell metabolism in muscle and liver cells. Sci. Rep..

[23] Goossens G.H. (2017). The metabolic phenotype in obesity: Fat mass, body fat distribution, and adipose tissue function. Obes. Facts.

[24] Swinburn B.A., Ley S.J., Carmichael H.E., Plank L.D. (1999). Body size and composition in Polynesians. Int. J. Obes..

[25] Rush E.C., Freitas I., Plank L.D. (2009). Body size, body composition and fat distribution: Comparative analysis of European, Maori, Pacific Island and Asian Indian adults. Br. J. Nutr..

[26] McAuley K.A., Williams S.M., Mann J.I., Goulding A., Murphy E. (2002). Increased risk of type 2 diabetes despite same degree of adiposity in different racial groups. Diabetes Care.

[27] Tiebe M., Lutz M., De La Garza A., Buechling T., Boutros M., Teleman A.A. (2015). REPTOR and REPTOR-BP regulate organismal metabolism and transcription downstream of TORC1. Dev. Cell.

[28] Burden H.J., Adams S., Kulatea B., Wright-McNaughton M., Sword D., Ormsbee J.J., Watene-O’Sullivan C., Merriman T.R., Knopp J.L., Chase J.G. (2021). The CREBRF diabetes-protective rs373863828-A allele is associated with enhanced early insulin release in men of Māori and Pacific ancestry. Diabetologia.

[29] Neel J.V. (1962). Diabetes Mellitus: A “thrifty” genotype rendered detrimental by “progress”?. Am. J. Hum. Genet..

[30] Kanshana J.S., Mattila P.E., Ewing M.C., Wood A.N., Schoiswohl G., Meyer A.C., Kowalski A., Rosenthal S.L., Gingras S., Kaufman B.A. (2021). A murine model of the human CREBRFR457Q obesity-risk variant does not influence energy or glucose homeostasis in response to nutritional stress. PLoS ONE.

[31] Reitman M.L. (2018). Of mice and men—Environmental temperature, body temperature, and treatment of obesity. FEBS Lett..

[32] Rossmeisl M., Rim J.S., Koza R.A., Kozak L.P. (2003). Variation in type 2 diabetes—Related traits in mouse strains susceptible to diet-induced obesity. Diabetes.

[33] Montgomery M.K., Hallahan N.L., Brown S.H., Liu M., Mitchell T.W., Cooney G.J., Turner N. (2013). Mouse strain-dependent variation in obesity and glucose homeostasis in response to high-fat feeding. Diabetologia.

